# Examining the performance of three ballast water compliance monitoring devices for quantifying live organisms in both regulated size classes (≥50 μm and ≥10–<50 μm)

**DOI:** 10.1093/plankt/fbad014

**Published:** 2023-05-14

**Authors:** Oscar Casas-Monroy, Torben Brydges, Jocelyn Kydd, Dawson Ogilvie, Robin M Rozon, Sarah A Bailey

**Affiliations:** GREAT LAKES LABORATORY FOR FISHERIES AND AQUATIC SCIENCES, FISHERIES AND OCEANS CANADA, 867 LAKESHORE ROAD, ON L7S 1A1, Canada; ST. ANDREWS BIOLOGICAL STATION, FISHERIES AND OCEANS CANADA, 125 MARINE SCIENCE DR, ST. ANDREWS, NB E5B 0E4, Canada; GREAT LAKES LABORATORY FOR FISHERIES AND AQUATIC SCIENCES, FISHERIES AND OCEANS CANADA, 867 LAKESHORE ROAD, ON L7S 1A1, Canada; GREAT LAKES LABORATORY FOR FISHERIES AND AQUATIC SCIENCES, FISHERIES AND OCEANS CANADA, 867 LAKESHORE ROAD, ON L7S 1A1, Canada; GREAT LAKES LABORATORY FOR FISHERIES AND AQUATIC SCIENCES, FISHERIES AND OCEANS CANADA, 867 LAKESHORE ROAD, ON L7S 1A1, Canada; GREAT LAKES LABORATORY FOR FISHERIES AND AQUATIC SCIENCES, FISHERIES AND OCEANS CANADA, 867 LAKESHORE ROAD, ON L7S 1A1, Canada

**Keywords:** ballast water management, phytoplankton, zooplankton, invasive species

## Abstract

A number of ballast water compliance monitoring devices (CMDs) have been made commercially available to verify the efficacy of ballast water management systems by quantifying the living organisms for both plankton size classes (≥50 μm and ≥10–<50 μm). This study aimed to examine whether new CMDs can provide a reliable indication of compliance regarding Regulation D-2 and to evaluate their performance for indicative analysis of organisms by assessing their accuracy (comparison to microscopy) and precision (comparison within measurement). Challenge fresh water samples were collected in four locations of Lake Ontario, Canada, whereas marine challenge water samples were collected around the Bay of Fundy, New Brunswick, Canada. Ballast water samples were collected from ships visiting several ports across Canada. Overall, accuracy was higher (>80%) in estimating organisms from prepared-challenge water (Ballast Eye and BallastWISE) than from ballast water samples (>70%) (B-QUA only). The sensitivity ranged from 50 to 100% for the ≥50 μm organism size class, whereas for the ≥10–<50 μm organism size class, it was higher for freshwater samples (>75%) than for marine samples (>50%). The performance of CMDs should be assessed under real-world conditions for a better understanding and to improve their use.

## INTRODUCTION

The release of ballast water is one of the most important vectors for the introduction of planktonic nonindigenous species (NIS). Once planktonic NIS are introduced to new environments, they can become established, causing negative effects on the functioning of aquatic ecosystems, and harm human health and economy (Ricciardi *et al*., 2013; Seebens *et al*., 2013; Bailey *et al*., 2020).

To minimize the introduction and spread of planktonic NIS, the International Convention for the Control and Management of Ship’s Ballast Water and Sediments (hereafter, BWM Convention) was adopted in 2004 and entered into force in September 2017 (International Maritime Organization, 2004). By 2024, the BWM Convention requires all ships to meet a performance standard (Regulation D-2) limiting the number of live organisms per unit of volume, which will typically be achieved by installing a type-approved ballast water management system (BWMS). The D-2 standard stipulates that ships shall discharge <10 viable organisms per m^3^ with a minimum dimension ≥50 μm (known as the “large” size class—typically zooplankton) and <10 viable organisms per mL with a minimum dimension ≥10 μm and <50 μm in minimum dimension (the “small” size class—typically phytoplankton). In addition, the D-2 standard stipulates discharge limits for certain microbial indicators (not studied in this research).

Various BWMS have been developed which typically use or combine different technologies—mechanical separation, chemical, or physical processes—to eliminate organisms. Ballast water is usually treated in a two-stage process, filtering large particles out to minimize sediments and abundance of large organisms before chemically or physically treating the water using active substances or ultra-violet light (Mouawad, 2015). BMWSs need to be tested and type approved following the Code for Approval of BWMSs (International Maritime Organization, 2018). Despite this requirement, it is acknowledged that BWMSs may not consistently meet the D-2 standard (Batista *et al*., 2017). Therefore, regulators need to have reliable methods to assess compliance with Regulation D-2, which is a critical component of management strategies to minimize the introduction of planktonic NIS.

Effective assessments of compliance with Regulation D-2 should result in collecting representative samples and to determine accurately the concentration of organisms in the two regulated size classes (≥50 μm and ≥10–<50 μm) inside ballast tanks. In recent years, several compliance monitoring devices (CMDs) have been made commercially available to verify the efficacy of BWMSs and assess compliance with Regulation D-2. These devices provide indicative estimates of live organism concentration that can be related to the D-2 standard, since direct counts using microscopy (standard reference method) are time consuming and require bulky, expensive equipment and scientific expertise (First and Drake, 2014). Initially, the majority of CMDs focused on quantifying the small plankton size class (Bradie *et al*., 2018; First *et al*., 2018; Vanden Byllaardt *et al*., 2018), relying on cellular attributes such as chlorophyll *a*, cell wall condition or enzymatic activity (Veldhuis and Kraay, 2000; Reavie *et al*., 2010; Peperzak and Brussaard, 2011). More recently, a limited number of CMDs quantify living organisms for both plankton size classes, relying on fluorescence signal, adenosine triphosphate (ATP) concentration or motility of organisms to estimate organism concentrations with presumptive similar accuracy to standard reference count methods.

For example, the Satake Ballast Eye Viable Organism Analyzer VOA 1000 K (hereafter Ballast Eye) uses pulse counting to detect fluorescence of organisms labeled with fluorescein diacetate (FDA) and converts the fluorescence signal to organism concentrations based on a conversion formula. The estimated organisms in m^3^ (for the ≥50 μm size class) or cells mL^−1^ (for the ≥10–<50 μm size class) are then reported as high risk (more viable organisms than the D-2 standard) or low risk (viable organisms less than or equal to the D-2 standard). The BallastWISE from MicroWISE can analyze both plankton size classes simultaneously or individually. The BallastWISE has two measuring chambers, one for each size class. The ≥50 μm size class chamber analyzes samples by tracking organism movement, whereas the ≥10–<50 μm size class chamber analyzes samples by both tracking organism movement and counting phototrophic organisms using an imaging-pulse amplitude modulation method. The estimated organism concentration is displayed with color code thresholds for compliance (green = very low risk, yellow = low risk and red = high risk), which is updated each time the chamber is refilled. Risks are defined by the manufacturer as “very low risk” having <13 organisms m^−3^ or cells mL^−1^, “low risk” having ≥13 to <30 organisms m^−3^ or cells mL^−1^ and “high risk” having ≥30 organisms m^−3^ or cells mL^−1^. Finally, the B-QUA from LuminUltra quantifies living organisms in the sample by measuring ATP concentration. The B-QUA uses reagents to extract the cellular ATP with an enzyme via cell lysis. Then the extract reacts with the luminase enzyme, and the active ATP is measured using a luminometer. LuminUltra has established compliance limits according to the BWM Convention for the two regulated organism size classes as well as total bacteria. The B-QUA reports units of ATP in pg m^−3^ (for the ≥50 μm size class) and pg mL^−1^ (for the ≥10–<50 μm size class). The results are interpreted using thresholds set by the vendor based on comparative studies with standard reference method counts performed on field samples reflecting a variety of organisms (LuminUltra, personal communication). For the ≥50 μm organism size class, compliance with the D-2 standard (i.e. <10 organisms m^−3^) is considered likely for <150 000 ATP (pg m^−3^), close to the D-2 limit between 150 000 and 750 000 ATP (pg m^−3^), and most likely not compliant with D-2 at > 750 000 ATP (pg m^−3^). For the ≥10–<50 μm organism size class, compliance with the D-2 standard is considered likely for <500 ATP (pg mL^−1^), close to the D-2 limit between 500 and 1500 ATP (pg mL^−1^), and most likely not compliant with D-2 at >1500 ATP (pg mL^−1^).

This study aimed to evaluate the performance of three new commercially available CMDs for indicative analysis of live organisms in the two regulated plankton size classes (≥50 μm and ≥10–<50 μm) in comparison to microscopy. Newer devices (Ballast Eye and BallastWISE) were tested using laboratory-prepared-challenge water collected from fresh and marine natural water sources covering a range of abundances and organism assemblages, whereas B-QUA (first device able to analyze the 2 regulated organism size classes) was only tested with ballast water samples collected from ships arriving at ports in Canada and prepared-challenge water samples from natural freshwater sources, due to its longer analysis time and higher consumables cost per measurement. We examined whether these devices can indicate compliance with the D-2 standard, regardless of the plankton communities contained in ballast water (fresh or marine water distribution). Furthermore, we examined whether the presence of these communities affects the accuracy and precision of the devices. The accuracy was assessed by comparing each CMD to the accepted standard reference method (bright-field microscopy based on motility (for the ≥50 μm size class) and epifluorescence microscopy based on FDA vital marker (for the ≥10–<50 μm size class). Precision was assessed based on the variation across measurements of prepared-challenge water subsamples (fresh and marine water).

## METHODS

### Laboratory tests—sample collection

**Fig. 1:**
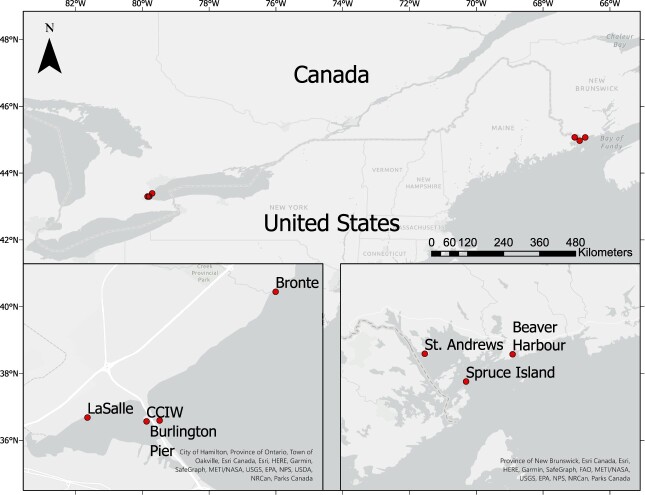
Sample locations in the Great Lakes, Ontario and the Passamaquoddy Bay region (Bay of Fundy), New Brunswick, Canada. Four locations were selected to collect ambient fresh water in the Great Lakes, whereas three locations were selected to collect ambient marine water in the Passamaquoddy Bay region. Ambient water was used to prepare challenge water to test each CMD at three concentration levels (low, medium and high).

Seven independent experiments were conducted during two consecutive field seasons (July–October). Ambient water from fresh water sources was collected from piers within Hamilton Harbor (2 locations) and the western part of Lake Ontario (2 locations), whereas ambient water from marine sources was collected from three locations from the Passamaquoddy Bay region (Bay of Fundy), New Brunswick, Canada (Fig. 1). CMDs were tested in the laboratory using ambient water prepared at various concentration levels (hereafter prepared-challenge water) containing a mixture of aquatic organisms (natural assemblages) collected from fresh and marine natural water sources.

From both fresh and marine natural water sources, zooplankton samples (≥50 μm size class) were collected using a 30 cm diameter, 35 μm (50 μm diagonal) mesh conical plankton net. The plankton net was lowered to a maximum depth of ~12 m and retrieved by hand at a speed of ~0.5 to 1 m s^−1^. The volume of water filtered ranged from 150 to 1800 L depending on the anticipated concentration of organisms in the area. Samples were concentrated into a 35 μm codend and rinsed with 10 μm filtered water (from the location of sampling) into a 1 L Nalgene bottle (initial sample). The 1 L bottle was topped up to 1 L using 10 μm filtered water, and initial abundance estimation was conducted by microscopy on the same day.

Phytoplankton samples (≥10–<50 μm size class) were collected from the surface water using a 20 L bucket at the same time as the zooplankton samples. The volume of water collected ranged from 20 to 520 L depending on the anticipated concentration of organisms. The water was filtered through a 35 μm Nitex mesh sieve and concentrated on a 10 μm Nitex mesh sieve (Sefar Inc, Depew, New York, USA). Organisms collected on the 10 μm mesh were rinsed into a 1 L Nalgene bottle (initial sample) using 10 μm filtered water from the location of sampling. The 1 L bottle was topped up to 1 L using 10 μm filtered water and stored in the dark, at the ambient local water temperature at the time of collection for initial abundance estimation by microscopy the next day.

In addition, >50 L of water was collected at each location of sampling and filtered on 10 μm mesh. At the laboratory, the water was further filtered using a hydrophilic polysulfone 0.2 μm millipore size membrane capsule (Pall Corporation), stored overnight in the dark at the same temperature at the ambient local water temperature at the time of collection (6–23 ± 2°C) and used to prepare dilutions. Environmental water quality data (i.e. temperature, pH, dissolved oxygen, salinity and turbidity) were recorded at each sample site using a YSI EXO 2 handheld multiparameter sonde equipped with appropriate sensors (YSI—Xylem Brand, OH, USA).

### Laboratory tests—sample preparation (prepared-challenge water)

The initial sample (1 L bottle) for the ≥50 μm organism size class was processed to prepare samples for accuracy assessment. The concentrated initial sample was mixed by gentle inversion five times and five aliquots were counted to determine the concentration of live organisms. Aliquots were transferred into single channels of a Modified Bogorov counting chamber (Hydro-Bios Apparatebau GmbH, Germany) using an Eppendorf pipette with the pipette tip trimmed to a 5 mm opening to allow the capture and transfer of larger organisms. Before loading the channel, a 10 μL aliquot of 50 μm beads was added to the counting chamber as a size reference (Fluoro-Max; Thermo Scientific, Waltham, USA). Live zooplankton ≥50 μm in minimum dimension were enumerated by broad taxonomic groups under a Nikon SMz800N Zoom stereoscope under ×30–80 magnification using standard movement/response to stimuli techniques (NSF International, 2010). Generally, counts aimed for a maximum of 25 individuals per channel by increasing (maximum of 2.0 mL) or decreasing (minimum 0.5 mL) the volume of aliquots based on the number of individuals counted in the first 1 mL aliquot. Organism counts were converted to an ambient concentration (i.e. number of organisms per m^3^) based on the volume and fraction of sample counted.

Once the concentration of organisms in the initial sample was determined, 1 L challenge water samples were prepared at three nominal concentration levels: low (<10 organisms m^−3^), medium (~20 organisms m^−3^) and high (~150 organisms m^−3^), where the 1 L sample is meant to be representative of a 1 m^3^ sample of ballast water that has been condensed to 1 L for transport. To prepare the low and medium concentration level samples, live organisms were transferred individually from a 5 mL sample tray to a 1 L sample bottle using a modified glass Pasteur pipette and bulb; the broad taxonomic group (e.g. copepods, nauplii, rotifers, polychaetes, etc.) of each individual was recorded. To prepare the high concentration level samples, a calculated volume of the initial sample was added to each 1 L sample bottle to produce the targeted concentration level. All the 1 L prepared-challenge water samples were initially filled half full, before organisms were transferred in, and then topped up to 1 L with 0.2 μm filtered water from the sampling location once the process was completed.

The following day, the initial sample (1 L bottle) for the ≥10–<50 μm size class was examined using FDA as a vital marker, as described in Adams *et al*. (2014). Briefly, the initial sample was mixed by gentle inversion five times and a 5 mL aliquot of each subsample was transferred to a scintillation vial where 417 μL of FDA working solution was added and incubated in the dark for 10 min. Then 1 mL was placed in a Sedgewick-Rafter counting chamber (Wildlife Supply Company, Yulee, Florida, USA) and 1 μL of 10 and 50 μm beads (Fluoro-Max; Thermo Scientific, Waltham, USA) was added to the chamber as size references for counting organisms in the targeted size class, ≥10 and <50 μm in minimum dimension. Six 1 mL subsamples were observed using a Zeiss Axio Vert.A1 microscope (Carl Zeiss Canada, Ltd, Toronto, Ontario, Canada), equipped with an LED Module (470 nm) and filter for green fluorescent protein (filter set 38, excitation 495 nm, emission 517 nm), and all fluorescing organisms (e.g. autotrophs, heterotrophs or mixotrophs) were counted in the entire chamber within 20 min. The 1 L initial sample was stored in the dark, and the temperature was maintained at the ambient local water temperature at the time of collection throughout the analysis.

The challenge water samples for the ≥10–<50 μm size class were prepared by adding a calculated volume of the initial sample and topped up with 0.2 μm filtered water from the respective sampling location to produce the organism concentrations of interest. Challenge water samples were prepared at three concentration levels: low (<10 cells mL^−1^), medium (~20 cells mL^−1^) and high (~150 cells mL^−1^). Three challenge water samples were prepared at each concentration level (low, medium, high) for analysis by each CMD and one for analysis by microscopy. Once the experiment was completed, the remaining volume from one of the high concentration level samples was preserved for taxonomic assessment by collecting a 125 mL subsample in a brown Nalgene bottle and adding 1.75 mL of Lugol’s acid. All initial and prepared-challenge water samples were stored in a dark, insulated container at the ambient local water temperature (6–23 ± 2°C) until analysis to minimize the mortality of organisms.

### Laboratory tests—accuracy experiments

The prepared-challenge water samples (low, medium and high concentration levels) were analyzed in parallel using the CMDs and microscopy as a standard reference method to estimate the concentration of live organisms for both size classes (≥50 μm and ≥10–<50 μm). For the ≥50 μm organism size class, one analyst counted one sample per concentration level using microscopy, whereas one analyst was assigned to each CMD to analyze one sample per concentration level. For the ≥10–<50 μm organism size class, two analysts counted two subsamples per concentration level using epifluorescence microscopy, whereas one analyst was assigned to each CMD to analyze one sample per concentration level. The analyst assigned to the Ballast Eye analyzed three subsamples as recommended by the manufacturer. For both size classes, challenge water samples were randomly assigned to each analyst, using a blind format where the concentration levels were unknown to the analysts.

### Microscopy

On the same day as sample collection, the 1 L prepared-challenge water samples for the ≥50 μm organism size class were concentrated to ~25 mL. Aliquots of 2 mL were counted in a modified Bogorov chamber, and broad taxonomic group was recorded. Samples were analyzed with the Nikon stereoscope as described previously, using the standard movement/response to stimuli techniques (NSF International, 2010). Counts were summed across aliquots and converted to ambient concentration (i.e. number of live organisms m^−3^). On the following day, prepared-challenge water samples for the ≥10–<50 μm size class were analyzed using FDA as a vital marker, as described previously. Four 1 mL subsamples of each concentration level were analyzed by two analysts using two Zeiss Axio Vert.A1 microscopes. The concentration of organisms in each prepared-challenge water sample (cells mL^−1^) at each concentration level was estimated as an average across the four subsamples.

### CMDs

The Ballast Eye and the BallastWISE were used to analyze prepared-challenge water samples from natural fresh and marine water sources (for accuracy and precision experiments), whereas the B-QUA was used to analyze prepared-challenge water samples from fresh water sources, as well as ballast water samples from both fresh and marine water sources. The prepared-challenge water samples were inverted five times to ensure samples were well mixed before removing each aliquot. Typically, CMDs estimate the concentration of organisms using one subsample for each size class (first day of experiments with the ≥50 μm size class and second day with the ≥10–<50 μm size class), except for the Ballast Eye, for which the manufacturer’s protocol recommends to use three subsample for the ≥10–<50 μm size class. The requirement for calibration or cleaning was followed as recommended by the manufacturers. The Ballast Eye was calibrated with its calibration cell and a blank was run before each measurement. The BallastWISE does not require calibration; however, the tubing was cleaned with distilled water between measurements and VIRKON® at the end of each analysis. The B-QUA was also calibrated and assessed using negative controls to verify that chemicals were not contaminated. Table I summarizes the procedures to analyze samples for each device.

**Table I:** Summary procedural description of analysis methods used to assess natural water and ballast water samples

Analysis method	Size class	Device technology	Procedural description
Microscopy	≥50 μm	Movement/response	A concentrated volume was counted using a modified Bogorov counting chamber and a Nikon Zoom stereoscope under ×30–80 magnification as a sum value (organisms m^−3^).
	≥10–<50 μm	Epifluorescence + FDA	Six 1 mL samples were stained with FDA, and cells counted using a Sedgewick-rafter plate and an epifluorescence microscope with fluorescence excitation based on LED modules as a single sum value (cells mL^−1^).
Ballast Eye	≥50 μm	Pulse counting FDA	A 1 L sample was filtered on 35-μm mesh sieve and transferred to a 100 mL cuvette and stained with FDA for assessment based on fluorescence emitted by organisms. Results are displayed as organisms m^−3^ and risk (low or high)
	≥10–<50 μm	Pulse counting FDA	Three 1 mL (for freshwater) or three 5 mL (for marine) samples were stained with FDA and transferred to a 100-mL cuvette for assessment based on fluorescence emitted by organisms. Results are displayed as cells mL^−1^ and risk (low or high).
BallastWISE	≥50 μm	Movement/response	A minimum of 500 mL of sample was pumped directly to the chamber for assessment based on movement of organisms. Results are displayed as organisms m^−3^ and risk (very low, low or high)
	≥10–<50 μm	Movement/Fluorescence	A minimum of 200 mL of sample was pumped directly to the chamber for assessment based on movement and fluorescence emitted by organisms. Results are displayed as cells mL^−1^ and risk (very low, low or high).
B-QUA	≥50 μm	Bioluminescence	A minimum of 300 L of sample prepared (multi-step process to extract and concentrate ATP) in a sample tube for assessment based on bioluminescence produced by ATP molecules in presence of luciferase enzyme. Results are displayed as relative luminescence units which are converted to ATP (pg mL^−3^); ATP values are compared with limits in Regulation D-2 using thresholds set by the vendor.
	≥10–<50 μm	Bioluminescence	About 200 mL of sample prepared (multi-step process to extract and concentrate ATP) in sample tube for assessment based on bioluminescence produced by ATP molecules in presence of luciferase enzyme. Results are displayed as relative luminescence units which are converted to ATP (pg mL^−1^); ATP values are compared with limits in Regulation D-2 using thresholds set by the vendor.

### Laboratory tests—precision experiments

The precision of the Ballast Eye and BallastWISE was assessed to determine the reproducibility of their measurements. Precision experiments for the ≥50 μm size class were conducted the same day the samples were collected (fresh and marine natural water sources). Ten 1 L samples, each containing nine organisms from the initial sample, were prepared for analysis by each device for the ≥50 μm organism size class. To ensure only nine organisms were added to each bottle, the selected organisms were first placed in a small Petri dish (using a modified glass Pasteur pipette and bulb) and checked for accidental bycatch before being rinsed into a 1 L Nalgene bottle filled with 0.2 μm filtered water from the sampling location. The following day, the precision experiment samples for the ≥10–<50 μm size class were prepared by adding a calculated volume of well-mixed initial sample to 1 LNalgene bottles based on the organism concentration in the initial sample (see Sample preparation for the accuracy experiment for details). Then each 1 L Nalgene bottle was topped up with 0.2-μm filtered water to produce the concentration level of interest (~9 cells mL^−1^). Each prepared-challenge water sample (10 × 1 L bottles per device) was analyzed with the Ballast Eye and BallastWISE for each size class. The B-QUA was not assessed in these experiments.

### Ballast water tests—sample collection

During three consecutive field seasons (May–October, 2017–2019), the ballast water samples were collected opportunistically from ships arriving at various marine ports in Canada (Pacific and Atlantic regions) or operating within the freshwater Great Lakes and St. Lawrence River region. The ballast water samples included: (i) samples collected during discharge operations of treated ballast water; and (ii) samples of paired uptake (untreated water) and discharge (treated water) ballast water. The samples were collected using the in-line isokinetic continuous collection methods described in Casas-Monroy *et al*. (2022) and Bailey *et al*. (2022), following the procedures recommended by the International Working Group on Ballast and Other Ship Vectors (ICES/IOC/IMO WGBOSV, 2017) and the IMO (International Maritime Organization, 2008). A large volume sample collection device was used to collect and measure up to three cubic meters of treated ballast water for the analysis of viable organisms in the ≥50 μm size class. A 30 cm diameter net with a 35 μm (50 μm diagonal) mesh (Wildco-Science First, USA) was employed to concentrate each 1 m^3^ sample, with the net being submerged in ambient ballast water in a 75 L bin to prevent organism damage. The net was rinsed with 10 μm filtered ballast water to concentrate live organisms into the codend, for transfer into a 1 L Nalgene bottle for further analysis.

In addition, a small volume sample collection device integrated into the large volume sample collection device was used to collect 20–30 L of whole water into a carboy for analysis of the ≥10–<50 μm size class. Carboys were gently overturned at least five times to mix their content and the water was subsequently split for analysis by B-QUA (1 × 1 L sample) and epifluorescence microscopy (1 L sample) (Casas-Monroy and Bailey, 2021; Casas-Monroy *et al*., 2022). All sample bottles were kept in a dark, insulated container with ice packs wrapped in bubble wrap (samples kept near or below ambient ballast water temperature) and analyzed within 3–4 h.

### Ballast water tests—sample analysis

At the laboratory, ballast water samples for the ≥50 μm size class were mixed by gentle inversion five times and split for analysis by microscopy and estimation of live organisms with the B-QUA. For microscope analysis, the ballast water samples were examined using a Nikon SMz800N Zoom stereoscope at ×30–80 magnification using the standard movement/response to stimuli techniques (; as described previously). Zooplankton assemblages information was summarized by recording the number of live individuals by broad taxonomic group (e.g. copepods, nauplii, rotifers, polychaetes, etc.) (see Bailey *et al*., 2022 for details on sample enumeration). Live counts were used to calculate the concentration of live individuals per m^3^ for each ship (based on the cumulative count across taxa and net samples). The ≥10–<50 μm size class samples were examined using FDA as a vital marker and a Zeiss Axio Vert.A1 microscope (as described previously).

Concurrently, both size classes were analyzed using the B-QUA, which was the first device able to analyze the two regulated organism size classes by measuring active ATP. The other two devices became available following the initial ballast water test. Phytoplankton assemblages were determined from natural and ballast water samples preserved with Lugol’s acid. Phytoplankton preserved samples were used for enumeration and taxonomic identification (Utermöhl, 1958) and summarized by broad taxonomic group (i.e. *Chlorophyceae*, *Bacillariophyceae*, *Dinophyceae*, etc.). Only intact cells with clearly visible cell content were assessed (see Casas-Monroy and Bailey, 2021 for details on sample collection and processing).

### Statistical analysis

Statistical analyses were conducted separately for each CMD and microscopy for results obtained from laboratory and ballast water tests (data available upon request). All analyses were performed using R software (version 4.05, R Development Core Team, 2021). Organism concentrations (mean across replicates) measured by each CMD were compared with microscopy (as standard reference method) for both size classes. Accuracy was defined as the closeness of measurements to the standard reference method. The accuracy of CMDs was assessed using the confusion matrix recording the total number of measures for which each CMD agreed or disagreed with microscopy in relation to the D-2 standard. Precision was defined as the closeness of measurements to each other. Precision was only assessed for the Ballast Eye and BallastWISE by calculating the coefficient of variation (CV, %; where 1 is equivalent to 100%)—a relative measure of the variation across measurements obtained only in laboratory tests (Casas-Monroy *et al*., 2022). The CV was calculated by dividing the standard deviation by the mean organism concentration of samples (*n* = 10) measured by each device. The results were compared based on organism size class and water source (fresh vs. marine water).

Sensitivity (i.e. the proportion of positive values correctly identified) and specificity (i.e. the proportion of negative values correctly identified) of CMDs were calculated using a confusion matrix (Tharwat, 2018; Casas-Monroy *et al*., 2022), reporting the number of true-positive, true-negative, false-positive and false-negative values. Measurements from CMDs were compared with microscopy counts in relation to the D-2 standard for both regulated size classes, such that a true-positive was scored when both measurements were >10 organisms m^−3^ or >10 cells mL^−1^ and a true-negative was scored when both measurements were <10 organisms m^−3^ or <10 cells mL^−1^. A false-positive (type I error) was scored when a CMD reported a value greater than the D-2 standard when the microscopy count was lower than the D-2 standard, whereas a false-negative (type II error) was scored when a CMD reported a value lower than the D-2 standard when the microscopy count was greater than the D-2 standard.

Finally, a generalized linear model (GLM) was conducted to examine differences between the CMDs and microscopy as reference method, using zooplankton (organisms m^−3^) and phytoplankton (cells mL^−1^) concentrations as the dependent variable, water source and organism concentration level as main factors. Since B-QUA (pg m^−3^ or pg mL^−1^) and organisms concentrations (organisms m^−3^ or cells mL^−1^) have different units, both metrics were log-transformed to allow multiple comparisons. Results were averaged over water source and concentration levels of organisms, using a confidence level of 0.95. Pairwise comparisons were performed using the Tukey method for *P*-value adjustment.

## RESULTS

### Laboratory tests

According to the microscopy counts, the actual concentration of ≥50 μm size class organisms in the prepared-challenge water samples was close to the nominal (target) levels of <10, 20 and 150 organisms m^−3^ (Table II). For both fresh and marine challenge water samples, the actual number of ≥50 μm size class organisms in the high concentration samples was marginally lower than the target levels, having 139 ± 18 and 114 ± 20 organisms m^−3^, respectively. Conversely, the concentration of ≥10–<50 μm size class organisms tended to be higher than the target levels for both medium and high treatments. In particular, the actual concentration for the high concentration level during the marine tests was estimated as 234 ± 10 cells mL^−1^ by epifluorescence microscopy (Table II).

**Table II:** Summary of measurements (mean ± standard error) obtained to assess accuracy of three CMDs in comparison to microscopy (standard reference method) for the ≥50 μm and ≥10–<50 μm plankton size classes across prepared-challenge fresh (FW) and marine water (MW) at three nominal concentration levels: low (<10 organisms m^−3^ or cells mL^−1^); medium (~20 organism m^−3^ or cells mL^−1^); high (~150 organisms m^−3^ or cells mL^−1^), and ballast water (BW—discharge or BW—uptake). N = number of samples analyzed (≥50 μm/≥10–<50 μm) and NA = samples not assessed with that particular CMD

		B-QUA ATP		Microscopy		BallastWISE		BALLAST Eye	
		(pg m^−3^)	(pg mL^−1^)	(org. m^−3^)	(cells mL^−1^)	(org. m^−3^)	(cells mL^−1^)	(org. m^−3^)	(cells mL^−1^)
Sample type	*N*	≥50 μm	≥10–<50 μm	≥50 μm	≥10–<50 μm	≥50 μm	≥10–<50 μm	≥50 μm	≥10–<50 μm
BW–Dis-MW	20/21	5.2E+06 ± 2.9E+06	14 ± 6.6	449 ± 260	1 ± 0.26	NA	NA	NA	NA
BW–Up-FW	7/7	5.0E+07 ± 1.3E+07	258 ± 74	46 721 ± 16 745	19 ± 7	NA	NA	NA	NA
BW–Dis-FW	7/7	1.0E+07 ± 9.9E+06	77 ± 47	159 ± 115	1 ± 0.8	NA	NA	NA	NA
Lab-Low-FW	4/4	4.7E+04 ± 1.1E+04	124 ± 45	3 ± 1.0	5 ± 1.0	9 ± 6	3 ± 1	5 ± 2	34 ± 15
Lab-Med-FW	4/4	1.0E+05 ± 2.4E04	179 ± 60	25 ± 6.0	30 ± 5	29 ± 10	8 ± 2	31 ± 6	60 ± 19
Lab-High-FW	4/4	2.6E+06 ± 7.1E+05	321 ± 73	139 ± 18	168 ± 16	492 ± 280	62 ± 22	269 ± 36	438 ± 120
Lab-Low-MW	3/3	NA	NA	2 ± 0.3	10 ± 0.2	0	2 ± 1	3 ± 1	2 ± 1
Lab-Med-MW	3/3	NA	NA	20 ± 1	41 ± 1	9 ± 9	13 ± 5	26 ± 3	7 ± 3
Lab-High-MW	3/3	NA	NA	114 ± 20	234 ± 10	80 ± 28	107 ± 23	147 ± 61	81 ± 47

The Ballast Eye produced measurements above the D-2 standard at the low organism concentration for the ≥10–<50 μm size class for prepared-challenge water samples from natural fresh water source, compared with microscopy. The BallastWISE produced measurements in agreement with microscopy for all but one trial at the low concentration level for both organism size classes (based on the manufacturer thresholds) for both fresh and marine prepared-challenge water samples. The B-QUA’s ATP measurements showed similar trends to microscopy for prepared-challenge water samples from fresh water sources, indicating that the B-QUA correctly identified samples that were above the D-2 standard for the ≥50 μm size class based on the manufacturer threshold; for the ≥10–<50 μm organism size class, the B-QUA produced measurements that were above the D-2 standard for the high and medium concentration levels based on the manufacturer threshold (Table II). The B-QUA was not assessed using prepared-challenge water samples from marine sources.

### Ballast water tests

Microscopy counts for marine ballast water discharge samples for the ≥50 μm organism size class ranged from 0 to 3822 organisms m^−3^ (*n* = 20, median = 11, mean = 449), with 10 samples exceeding the D-2 standard (15–3822 organisms m^−3^, median = 71). Epifluorescent microscopy counts for the ≥10–<50 μm organism size class ranged from 0 to 5 cells mL^−1^ (*n* = 21, median = 1, mean = 1) (Table II).

For uptake and discharge freshwater samples, microscopy counts for the ≥50 μm organism size class ranged from 2168 to 107 577 organisms m^−3^ (*n* = 7, median = 25 159, mean = 46 721), and from 0 to 803 organisms m^−3^ (*n* = 7, median = 4, mean = 159), respectively; three discharge samples exceeded the D-2 standard (12–803 organisms m^−3^, median = 408). For uptake and discharge samples, epifluorescent microscopy counts for the ≥10–<50 μm organisms size class ranged from 3 to 57 cells mL^−1^ (*n* = 7, median = 12, mean = 19) and from 0 to 1 (*n* = 7 median = 1, mean = 1), respectively; three out of seven uptake samples were below the D-2 standard.

The B-QUA showed values ranging from 3.49E+3 ATP pg m^−3^ to 5.65E+07 ATP pg m^−3^ for marine ballast water discharge samples for the ≥50 μm organism size class, identifying 14 out of 20 samples as exceeding the D-2 standard. For the ≥10–<50 μm organism size class, the B-QUA produced no measurements above the D-2 standard (*n* = 21). Considering uptake and discharge freshwater samples for the ≥50 μm size class, the B-QUA estimation ranged from 1.08E+07 to 8.88E+07 ATP pg m^−3^ (*n* = 7, median = 3.87E+07, mean = 5.04E+07) and from 6.23E+04 to 6.98E+07 ATP pg m^−3^ (*n* = 7, median = 9.38E+04, mean = 1.04E+07), respectively; all seven uptake samples exceeded the D-2 standard, whereas two out of seven discharge samples exceeded the D-2 standard. For the ≥10–<50 μm organism size class, the B-QUA indicated that only one freshwater uptake sample exceeded the D-2 standard, whereas the remaining uptake and discharge freshwater samples (13) were below the D-2 standard (Table II).

### Community composition

Identification of broad taxonomic groups for the ≥50 μm size class showed that nauplii, rotifers and copepods dominated freshwater laboratory experiments samples with a relative abundance ranging from 10 to 83%, 5 to 42% and 2 to 23%, respectively. Nauplii, copepods and rotifers also dominated marine laboratory experiments with a relative abundance ranging from 50 to 58%, 8 to 40% and 2 to 13%, respectively (Supporting Information Table S1). Overall, freshwater experiments were conducted with at least five different families of rotifers and three orders of copepods.

For the ≥10–<50 μm organism size class, *Cyanophyceae*, *Chlorophyceae*, *Bacillariophyceae* and *Alveolata* dominated freshwater laboratory experiments with a relative abundance ranging from 7 to 45%, 26 to 72%, 0.1 to 30% and 13 to 45%, respectively (Supporting Information Table S1). *Bacillariophyceae* and *Alveolata* also dominated marine laboratory experiments with a relative abundance ranging from 42 to 80% and 20 to 58%, respectively. At least 2 *Cyanophyceae*, 7 *Chlorophyceae*, 11 *Bacillariophyceae* and 3 *Alveolata* species were identified in samples from freshwater experiments, whereas at least 11 *Bacillariophyceae* and 13 *Alveolata* species were identified in marine experiments.

Broad taxonomic groups for the ≥50 μm and the ≥10–<50 μm organism size classes were identified in ballast water from ships visiting the Pacific coast of Canada and the Great Lakes region. For the ≥50 μm organism size class, nauplii, copepods and rotifers dominated marine ballast water samples with a relative abundance ranging from 0 to 100%, 0 to 100% and 0 to 71%, respectively (Supporting Information Table S1). Copepods were found in 62% of the samples, whereas nauplii and larvae (i.e. Veligers) were present in 57 and 33% of the samples, respectively. Copepods, rotifers and nauplii dominated fresh ballast water samples with a relative abundance ranging from 0 to 100%, 0 to 95% and 0 to 65%, respectively. Nauplii were found in 86% of the samples, whereas Diplostracans (formely Cladocerans), rotifers and copepods were present in 79% of the fresh ballast water samples.

For the ≥10–<50 μm organism size class, *Bacillariophyceae* and *Chlorophyceae* dominated the marine ballast water samples with a relative abundance ranging from 0 to 100%. *Bacillariophyceae* were found in 95% of the samples, *Chlorophyceae* in 38%, whereas *Alveolata* were present in 86% of the samples.

*Bacillariophyceae* , *Cyanophyceae* and *Chlorophyceae* dominated fresh ballast water samples with a relative abundance ranging from 0 to 95%, 0 to 68% and 0 to 64%, respectively. *Bacillariophyceae* and *Chlorophyceae* were found in 100% of the samples, whereas *Cyanophyceae* were present in half of the samples, regardless of ballast operations (uptake or discharge). *Dictyophyceae* were found in 71% of the samples, although the relative abundance only ranged from 0 to 17%.

### Accuracy

For prepared-challenge water samples, the Ballast Eye had 100% accuracy estimating the concentration of organisms for the ≥50 μm size class in fresh and marine natural water samples, but had lower accuracy for the ≥10–<50 μm organism size class in these samples (80 and 60%, respectively). BallastWISE had high accuracy (80%) for the ≥50 μm size class in fresh and marine natural water samples, whereas for the ≥10–<50 μm organism size class, it was higher for fresh water (80%) compared with marine natural water samples (60%). The B-QUA had higher accuracy (70%) for the ≥50 μm organism size class than the ≥10–<50 μm organism size class in fresh natural water samples (33%) (Table III).

**Table III:** Summary of accuracy of CMDs in comparison to microscopy (standard reference method) for the ≥50 μm and ≥10–<50 μm plankton size classes during laboratory and ballast water tests, where Agreement (AG) = measurement in accordance with microscopy; Disagreement (DISAG) = measurement in discordance with microscopy; True positive (TP) = a value greater than the D-2 standard, in agreement with microscopy; True negative (TN) = a value lower than the D-2 standard, in agreement with microscopy; False-negative (FN) = a value lower than the D-2 standard, in disagreement with microscopy; False-positive (FP) = a value higher than the D-2 standard, in disagreement with microscopy; Accuracy = TN + TP/TP + TN + FP + FN; Sensitivity = TP/TP + FN; Specificity = TN/TN + FP. NA = a measurement that could not calculated due to zero values (division by zero is not possible)

Size class	CMD	Sample type	Water source	Total samples	AG (%)	DISAG (%)	TP	TN	FN	FP	Accuracy (%)	Sensitivity (%)	Specificity (%)
≥50 μm	Ballast Eye	Lab	Freshwater	12	1.0	0.0	8	4	0.0	0.0	1.0	1.0	1.0
		Lab	Marine	9	1.0	0.0	6	3	0.0	0.0	1.0	1.0	1.0
	BallastWISE	Lab	Freshwater	12	0.8	0.2	7	3	0.1	0.1	0.8	0.9	0.8
		Lab	Marine	9	0.8	0.2	4	3	0.2	0.0	0.8	0.7	1.0
	B-QUA	Lab	Freshwater	12	0.7	0.3	4	4	0.3	0.0	0.7	0.5	1.0
		BW	Marine	20	0.9	0.2	9	8	0.1	0.1	0.9	0.9	0.8
		BW	Freshwater	14	0.9	0.1	9	4	0.1	0.0	0.9	0.9	1.0
													
≥10–<50 μm	Ballast Eye	Lab	Freshwater	12	0.8	0.3	8	1	0.0	0.3	0.8	1.0	0.3
		Lab	Marine	9	0.6	0.4	4	1	0.4	0.0	0.6	0.5	1.0
	BallastWISE	Lab	Freshwater	12	0.8	0.2	6	4	0.2	0.0	0.8	0.8	1.0
		Lab	Marine	9	0.6	0.4	4	1	0.4	0.0	0.6	0.5	1.0
	B-QUA	Lab	Freshwater	12	0.3	0.7	0	4	0.7	0.0	0.3	NA	1.0
		BW	Marine	21	1.0	0.0	0	21	0.0	0.0	1.0	NA	1.0
		BW	Freshwater	14	0.8	0.2	1	10	0.2	0.0	0.8	0.3	1.0

For ballast water samples, accuracy was only assessed for the B-QUA. Overall, accuracy ranged from 80 to 100% for both organism size classes, regardless of the ships’ ballast water operation: discharge (marine samples) or uptake/discharge (freshwater samples).

### Sensitivity and specificity

Sensitivity and specificity of the CMDs were assessed using prepared-challenge water samples by calculating the number of false-negative and false-positive values. For the ≥50 μm organism size class, the Ballast Eye had high sensitivity (100%) for fresh and marine water samples given the absence of false-negative values. The BallastWISE had higher sensitivity for freshwater samples (88%) than marine samples (67%) due to a lower number of false-negative in freshwater samples (1) than marine samples (2). The B-QUA had low sensitivity for freshwater samples (50%) given its high number of false-negative (4). For the ≥10–<50 μm organism size class, the Ballast Eye and BallastWISE had higher sensitivity for freshwater samples (100 and 75%, respectively) than marine samples (50%). Sensitivity could not be calculated for the B-QUA for the ≥10–<50 μm organism size class due to zero values (division by zero is not possible).

The Ballast Eye had high specificity (100%) for the ≥50 μm organism size class for both fresh and marine water samples but had low specificity (30%) for the ≥10–<50 μm organism size class in fresh water samples. The specificity of BallastWISE was higher than 80% for both organism size classes, regardless if samples were fresh or marine water.

Sensitivity and specificity of the B-QUA were also assessed based on the ballast water samples using the number of false-negative and false-positive values. The sensitivity of the B-QUA was high (90%) for the ≥50 μm organism size class for all ballast water samples (marine and fresh water samples). On the other hand, the B-QUA had poor sensitivity (25%) for the ≥10–<50 μm organism size class for uptake and discharge samples due to the rate of false-negative values. Finally, the B-QUA had high specificity (>80%) for all ballast water samples for both organism size classes, regardless of the ships’ ballast water operation (discharge or uptake/discharge) and ballast water source (fresh or marine water) (Table III).

### Precision

**Fig. 2:**
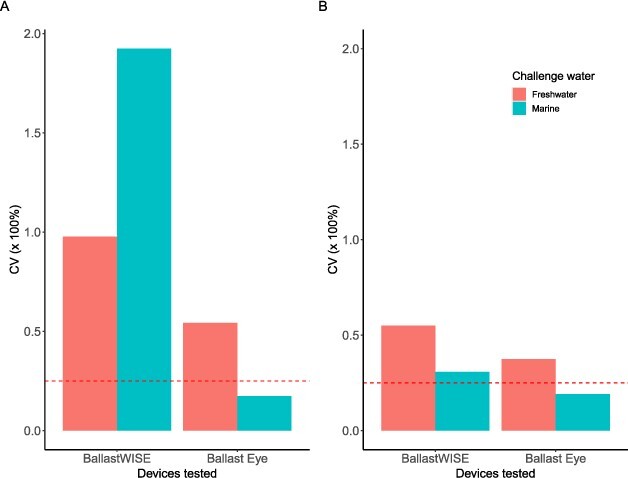
CV for the ≥50 μm organism size class (left panel-A) and ≥10–<50 μm organism size class (right panel-B) calculated based on the analysis of 10 laboratory samples for the BallastWISE and Ballast Eye. The horizontal red dashed line indicates CV = 0.25.

For prepared-challenge water samples, the CV ranged from 0.17 to 1.92 for the ≥50 μm organism size class and 0.19 to 0.55 for the ≥10–<50 μm organism size class (Fig. 2). The Ballast Eye had a lower CV than 0.25 for the ≥50 μm organism size class for both fresh and marine samples and for both organism size classes for marine samples. The BallastWISE had a higher CV for the ≥50 μm organism size class than the ≥10–<50 μm organism size class for both fresh and marine samples. Precision was not assessed for the B-QUA.

### Statistical results

For prepared-challenge water samples, the GLM model showed that the CMDs were not significantly different than microscopy in estimating the categorical organism concentrations for the ≥50 μm size class for the same concentration level (e.g. high, medium or low) for both fresh and marine water sources. The ATP estimates by B-QUA were significantly higher for all three concentration level compared with microscopy (Estimate = 9.5; Std. Error = 0.7; t-ratio = 16; *P* < 0.0001). The GLM model showed no differences between CMDs and epifluorescence microscopy in estimating the categorical organism concentrations for the ≥10–<50 μm size class for the same concentration level (e.g. high, medium or low) for both fresh and marine water sources. The ATP estimates by B-QUA were significantly higher compared with epifluorescence microscopy at the low concentration level (Estimate = 2.8; Std. Error = 0.6; t-ratio = 5.2; *P* < 0.0009), whereas no differences were observed for the other two concentration levels (high and medium).

For ballast water samples, the GLM model showed that B-QUA had significantly lower ATP estimates for the ≥50 μm organism size class in discharge samples compared with uptake samples (Estimate = −6.1; Std. Error = 0.9; t-ratio − 6.4; *P* < 0.0001); similar trends were observed between discharge and uptake samples for the ≥10–<50 μm organism size class. Overall, trends were similar to microscopy (for the ≥50 μm organism size class) and epifluorescence microscopy (for the ≥10–<50 μm organism size class), regardless of the ballast water source (marine or freshwater).

## DISCUSSION

The focus of this research was to determine if CMDs can be used to analyze ballast water to estimate whether the concentration of live organisms exceeds the D-2 standard. The CMDs examined were able to detect live organisms in both regulated size classes (≥50 μm and ≥10–<50 μm), produce numerical values and indicate pass, fail or NA (when values were below or above the detection limit). The CMDs appropriately categorized most discharge ballast water and prepared-challenge water samples as low, medium or high concentration of organisms. Nevertheless, the CMDs also produced a number of false-positive and false-negative rates for both size classes probably due to the inherent variability of natural assemblages of organisms.

Studies have recently examined the benefits and drawbacks of using CMDs for ballast water management measuring living organisms (in m^3^ or mL) using ballast water or prepared-challenge water samples (Drake *et al*., 2014; First et al., 2018; Casas-Monroy *et al*., 2022). However, results are more reliable when samples are categorized as pass, fail or not determined. In so doing, analysis of samples and interpretation of results could be beneficial to non-scientist personnel such as vessel inspectors, BWMS commissioning test teams, Port State control officers, ship owners and crew, by providing timely, accurate and reliable data.

Although CMDs may report numerical values or estimates of live organism concentrations, most are designed to detect exceedances of discharge limits, without necessarily exhibiting a linear response compared with the reference method. Therefore, accuracy and precision were calculated based on the agreement between the CMD and the reference method (i.e. microscopy based on motility for the ≥50 μm size class or epifluorescence for the ≥10–<50 μm), resulting in a number of false-negative and false-positive rates. Most of the false-negative and false-positive rates observed in this present study were likely due to some CMDs having high thresholds compared with levels near (or below) the limit of the D-2 standards, particularly for the ≥10–<50 μm size class. Similar findings have been reported by McClung *et al*. (2021), where the Ballast Eye was unable to accurately estimate the concentration of ambient organisms within the ≥10–<50 μm size class in water from Lake Superior. The mismatch between the results of the Ballast Eye and the reference method could also be due to the community composition in a given aquatic environment, as well as differences between the manufacturer’s sample processing protocols and those used in scientific studies.

Our results showed similar trends between the CMDs and microscopy counts across concentration levels for both the ≥50 μm and ≥10–<50 μm size classes, regardless of whether samples were marine or fresh water. For the ≥50 μm size class, no differences were observed between the CMDs and microscopy for freshwater samples, whereas BallastWISE estimated fewer organisms in marine samples than microscopy, probably due to differences in zooplankton community. Freshwater samples were dominated by small taxa (i.e. >70% nauplii + rotifers), whereas marine samples were dominated by large taxa (>40% copepods and tunicates). These differences may have lowered the accuracy of BallastWISE when processing marine samples possibly due to the escape behavior of larger organisms to avoid the tubing and be counted by the instrument. For the ≥10–<50 μm size class, the Ballast Eye did not accurately estimate low organism concentrations in freshwater samples and estimated fewer organisms in marine samples than microscopy. Our community composition analysis revealed that freshwater samples were dominated by *Chlorophyceae* and *Cyanophyceae* (groups with large number of species capable of producing colonies with cells frequently measuring >10 μm), whereas marine samples were mostly dominated by diatoms and dinoflagellates (cells frequently measuring >10 μm).

Tests conducted using live organisms of *Scenedesmus quadricauda* found that the Ballast Eye did not accurately detect moderate to high concentrations of colonial organisms in a sample when cells forming colonies are smaller than 10 μm in minimum dimension (McClung *et al*., 2021); however, this study noted that results could be affected due to a failure of the vital marker to effectively stain cells. Interestingly, our results showed that the Ballast Eye accurately estimated organism concentrations in marine samples that had high number of diatoms forming chains. These chains of diatoms were likely broken by the device’s stirring motion, facilitating estimation due to larger individual cell sizes than the cells in colonies in freshwater samples. A previous examination of the BallastWISE reported that the probability of detecting an exceedance of the D-2 standard was not likely (>0.5 probability) until a concentration of 17 cells mL^−1^ (Nagel *et al*., 2021); this higher value may partly be due to the device’s ability to distinguish between cells and colonies. The differences in community composition observed in this study may have an impact on measurement accuracy. For example, the Ballast Eye had high accuracy (>95%) in estimating marine and fresh water organisms for the ≥50 μm size class but low accuracy (<75%) in estimating marine and fresh water organisms for the ≥10–<50 μm size class, whereas the BallastWISE had higher accuracy (>83%) in estimating fresh water organisms from both size classes compared with marine organisms.

Discrepancies between CMDs and the reference method were a mix of false-negative and/or false-positive results for both organism size classes. Although all three CMDs were tested using prepared-challenge water samples from fresh water natural sources in laboratory tests, only the Ballast Eye and BallastWISE were tested using prepared-challenge water samples from marine natural sources, whereas the B-QUA was tested on marine and fresh ballast water samples. The Ballast Eye had similar trend to microscopy for the ≥50 μm size class; however, it produced false-positive (~0.3) and false-negative (~0.4) rates at the lowest concentration level in freshwater and marine samples, respectively, for the ≥10–<50 μm size class, mismatching low measurements against the reference method; although based on the latter, prepared-challenge water samples from marine sources were around the D-2 standard (~10 cells mL^−1^).

The BallastWISE produced some tracking errors, generally caused by air bubbles, even when the device was operated on a level surface in a vibration-free environment. All prepared-challenge water samples were appropriately categorized as very low, low or high risk by the device, but some work was required to correctly analyze blank samples as containing no organisms. Nevertheless, the BallastWISE produced false-negative rates at the medium concentration level in marine samples for the ≥50 μm size class (~0.2). The B-QUA had a high percentage of agreement with microscopy measuring the ≥50 μm organism size class, and also higher percentage of agreement measuring organisms’ ATP in marine samples (90%) than in prepared fresh water or ballast water samples (50%). These discrepancies between the B-QUA and the reference methods may be due to the low sensitivity of the instrument measuring ATP from samples with high number of small organisms (i.e. ~10 μm; LuminUltra comp. Pers.), particularly in uptake ballast water.

The results of this study may have been influenced by variations in the CMD protocols for sample processing. All three CMDs were utilized following manufacturer’s protocols and specifications. The CMDs were able to operate within the environment they have been designed for use in, and they assessed the groups of organisms they were intended to quantify based on manufacturer’s recommendations. Some issues were encountered with the software of the BallastWISE and calibration process of the Ballast Eye, which were resolved after receiving technical assistance from their manufacturers. Special care was also taken with sample temperature to avoid issues that may impact the Ballast Eye results. The protocol called for reagents and samples to be kept at a minimum temperature of 21°C. However, depending on the season, it may be difficult to maintain 21°C without stressing the organisms, which may affect the instrument’s readings. This was observed in one of our trials that was not included in this analysis.

## CONCLUSIONS

All CMDs examined in this study estimated living organisms facilitating evaluation with the D-2 standard. Although these CMDs are technological advancements in aquatic organism counting and are relatively easy to operate, some basic knowledge about species biology, and the sample conditions (i.e. salinity, temperature), is required to minimize user error and facilitate data interpretation.

Each of the three CMDs tested here has benefits and drawbacks that must be considered when deciding which CMD to use for monitoring compliance with the D-2 standard. For example, the B-QUA protocol is lengthy and data cannot be directly compared with numerical values from microscopy. However, ATP appears to be effective (sensitive) for detection of living organisms in samples. The Ballast Eye performed well with marine and fresh natural water samples, accurately estimating the ≥50 μm organism size class. However, this CMD did not accurately estimate the low organism concentrations level in fresh and marine prepared-challenge water samples during this study. The BallastWISE does not use chemicals to analyze either of the two organism size classes. However, the BallastWISE produced tracking errors (usually caused by air bubbles) when assessing the ≥50 μm organism size class, even though it was operated on a level surface in a vibration-free environment. Overall, a CMD capable of analyzing both organism size classes is a significant step forward in confirming ships’ compliance with discharge D-2 standard. It would be ideal to test the Ballast Eye and the BallastWISE with ballast water samples to better understand how these CMDs work under real-world conditions to confirm their performance.
